# Environment and social support for smoking cessation among community smokers in Beijing, China

**DOI:** 10.18332/tid/172216

**Published:** 2023-11-07

**Authors:** Mingyu Gu, Xingming Li, Tingting Qin, Kun Qiao, Xinyuan Bai, Yao Wang, Yutong Yang, Yu Bai, Jie Gao

**Affiliations:** 1 School of Public Health, Capital Medical University, Beijing, China

**Keywords:** environment support, social support, smoking cessation, secondary analysis, cross-sectional survey

## Abstract

**INTRODUCTION:**

This study investigated the relationship between environment support, social support and smoking cessation, to provide suggestions for the construction of environment and social support for tobacco control.

**METHODS:**

This was a secondary analysis based on two cross-sectional surveys of adult smokers who participated in the six-month community smoking cessation intervention projects in Beijing. The study subjects were divided into a successful group (n=159) and an unsuccessful group (n=253). The status of the environment support, community tobacco exposure, and social support were compared between groups. A structural equation model (SEM) was established for Confirmatory Factor Analysis.

**RESULTS:**

The univariate analysis showed that there were differences in smoking cessation outcomes among smokers with different home tobacco regulations, workplace tobacco regulations and number of smokers in the family. Differences in 6-month smoking reduction were also found with different promotion levels of anti-tobacco messaging and the frequency of smoking events at home. The logistic regression analysis indicated that home tobacco regulations (OR=1.30; 95% CI: 1.00–1.69), workplace tobacco regulations (OR=1.27; 95% CI: 1.05– 1.54), and frequency of smoking events at home (OR=1.15; 95% CI: 1.02–1.29), were associated with smoking cessation. The results of the SEM showed that environment support (β=0.39; 95% CI: 0.05–0.73, p=0.026) and social support (β=0.37; 95% CI: 0.05–0.68, p=0.022) had influence on smoking cessation.

**CONCLUSIONS:**

Environment support and social support are related to smoking cessation. Attention should be paid to the smoking regulations at home and workplace, anti-tobacco messaging, and social support by the family.

**TRIAL REGISTRATION:**

The study was registered on the official website of the China Clinical Trial Registration Center. Identifier: ChiCTR1900024991

## INTRODUCTION

Tobacco harm is one of the most serious public health problems in the world today^1^. According to the Global Burden of Disease Study 2019, the leading Level 2 risk factor globally for attributable deaths was tobacco, which accounted for 8.71 million deaths (15.4% of all deaths in 2019)^2^. According to the 2018 Chinese Adult Tobacco Survey results^3^, the smoking rate of people aged >15 years in China was 26.6% in 2018. Although 19.8% of smokers tried to quit smoking, 13% of the quitters relapsed within two years^4^. Chinese smokers have the willingness to quit smoking, but the success rate is low.

Common approaches to smoking cessation include clinical consultation, pharmacological therapy, nicotine replacement therapy, behavioral intervention, and multi-component smoking cessation treatment^5^. Previous studies have shown that health status, environmental support, tobacco harm knowledge, economic level, and doctor’s persuasion, affect smoking cessation^6^. It can be seen that the environment and social support for tobacco control can affect the smoking cessation behaviors. Environmental support refers to tobacco regulations within environments that promote smoking cessation. Social support refers to the care and cessation support that people feel they receive from other people^7^. The establishment of environment and social support for tobacco control has a continuous impact on smoking cessation behavior, acting as an important condition for smoking behavior changes^8,9^. Some studies have shown that the influence of the environment and social support for tobacco control can effectively promote smoking cessation among smokers^10-12^, while the proportion of other smokers in the environment and the number of tobacco advertisements are inversely correlated with smoking cessation outcomes^11,13,14^. Creating a 100% smoke-free environment and providing effective smoking cessation measures could improve smoking cessation, but which factors in the environment and social support for tobacco control determine smoking cessation outcomes and how these factors act, needs to be further clarified.

The study subjects were adult smokers who participated in a community smoking cessation intervention project in Beijing. We analyzed the relationship between environment support, social support and behaviors of smoking cessation among smokers, to provide suggestions for the construction of environment and social support for tobacco control.

## METHODS

This study is a secondary analysis based on two cross-sectional surveys (baseline survey and follow-up survey) of the National Key Research and Development Program of China. Using convenience sampling, we selected 19 community health service centers with stable population and chronic disease management as the survey objects in Beijing. Data were collected from December 2018 to December 2019^15^. Community doctors and community managers were responsible for recruiting smokers who were willing to participate in the smoking cessation programs and face-to-face interviews by trained investigators were used to obtain data for 683 participants^15^.

### Inclusion criteria

Those who were included^15^ were smokers who: 1) had smoked for ≥6 months and had smoked within 30 days prior to the survey, 2) had not currently used other methods to quit smoking, 3) could verbally communicate fluently, and 4) were willing to take part in the follow-up.

### Exclusion criteria

Those who were excluded^15^ were: 1) non-smokers, 2) smokers who had participated in another smoking cessation program, 3) reluctant to participate in smoking cessation programs, 4) pregnant or breast feeding, and 5) suffering from a serious illness that prevented them from being able to participate physically or mentally.

In this study, based on the cross-sectional survey, samples with an unknown smoking cessation outcome during the follow-up survey were excluded and the extreme values of the survey data were cleaned up. A total of 412 smokers were finally included in this study. The data processing is shown in Supplementary file Figure S1.

As the analytical approach is that of a case control study, 159 smokers were included in the case group who had quit smoking after 6 months of evaluation, and 253 smokers were included in the control group who had not quit smoking after evaluation. The environmental factors of smoking cessation behavior and outcome are analyzed from the perspective of health promotion through Social Ecological Theory (SET), which believes that smoking cessation behavior and outcome are affected by five levels: intrapersonal, interpersonal, organizational, community, and public policy^16^. A systematic review, with reference to SET, analyzed the factors that influence social ecological factors on nicotine replacement therapy and found that individual, public policy, community, and interpersonal factors can have an impact on smoking cessation behaviors and cessation outcomes^17^. We thus focused our attention on the influence of external environmental factors on smoking cessation. This study thus divided the survey data into three aspects: environment support, community tobacco exposure, and social support. The differences among smokers with a cessation outcome were compared and analyzed in terms of: explicit tobacco smoking regulations in the community, home, and workplace; exposure to anti-tobacco messaging; indoor and outdoor tobacco exposure in the community; the number of smokers in the family; the frequency of smoking events at home; and family support for smoking cessation.

The main variables used to assess smoking cessation outcome, environment support, community tobacco exposure, and social support were derived from survey data from a previous study^18^. The assessment of the environment and social support for tobacco control included latent and observed variables. An observed variable is one that can be directly observed, and a latent variable is usually one that cannot be directly observed and needs to be estimated with the help of an exogenous measure^19^. In this study, latent variables included: 1) smoking cessation, 2) environment support, 3) community tobacco exposure, and 4) social support. Observed variables included: 1) smoking cessation outcome and 6-month smoking reduction; 2) explicit tobacco regulations in the community, home and workplace, and anti-tobacco messaging in the community; 3) smoking is seen in indoor and outdoor places in the community; and 4) the frequency of smoking events at home, the number of smokers in the family, and whether family members support smoking cessation. The definition and measurement of each variable are shown in Supplementary file Table S1.

The cross-sectional survey used the following protocols to ensure data quality: 1) All survey were carried out by trained investigators using a uniformly designed questionnaire, with reviewers assessing the questionnaires and checking for gaps; 2) Double-entry of questionnaires using Epidata, proofreading inconsistent data one-by-one, and eliminating questionnaires of poor quality; 3) The participants were strictly reviewed according to the inclusion and exclusion criteria, and they were required to register by real name to ensure the authenticity of the information. Informed consent was obtained, and the privacy of the participants was ensured; and 4) A carbon monoxide breath test (Micro^+^ Smokerlyzer, bedfont) was used to measure the carbon monoxide concentration (ppm) exhaled to ensure the authenticity of their smoking cessation outcome.

### Statistical analysis

Epidata V 3.1 was used to organize and summarize the questionnaire data, and to establish and manage the database. The data were imported into SPSS 19.0 for processing and analysis. A chi-squared test, Fisher’s exact test, and a non-parametric test (Mann-Whitney U test) were conducted to analyze data on smokers’ demographic information and tobacco control interventions, to ensure comparability of data on the environment and social support for tobacco control between quitters and non-quitters. The qualitative variables obtained from the questionnaires were described, and the variables (community tobacco regulations, home tobacco regulations, workplace tobacco regulations, anti-tobacco messaging, community indoor tobacco exposure, and community outdoor tobacco exposure) were compared between groups using a chi-squared test to examine their relationship. In addition, variables with expected frequencies <5 (the frequency of smoking events at home, number of smokers in the family, and family support for smoking cessation) were compared between groups using Fisher’s exact test. The 6-month smoking reduction was a quantitative variable and did not conform to a normal distribution, so medians with interquartile range (IQR) were used with a non-parametric test (Kruskal-Wallis test) for comparisons between groups. Subsequent binary logistic regression analyses were used to explore the association of factors between groups with different smoking cessation outcomes.

**Figure 1:**
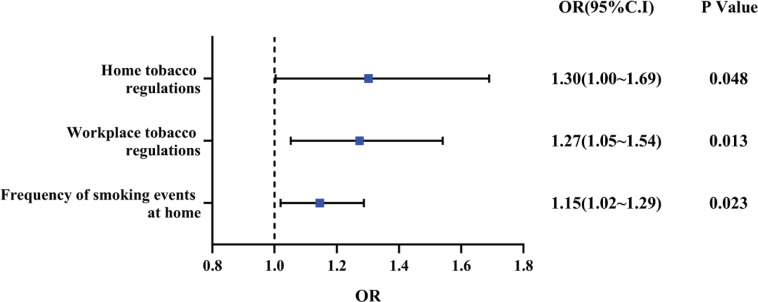
Binary logistic regression of smoking cessation outcomes, 2019 (N=412)

Using Mplus 8.3 we constructed a SEM (structural equation model) based on the study context and the investigated variables, and conducted Confirmatory Factor Analysis (CFA), with the WLSMV method for parameter estimation^19^. To improve model identification, samples with missing environment and social support variables of tobacco control were excluded from the 412 cases, and the final sample of 375 cases were included in the CFA of the SEM, as shown in Figure 1. All statistical analyses were 2-tailed and performed at a test level of α=0.05, a significant difference was considered for p<0.05.

## RESULTS

### Demographic information

There were 412 subjects included after validation of questionnaires and data cleaning. Among these, 374 (90.8%) were male, 125 (30.4%) were aged 50–59 years, 139 (33.8%) were aged 60–69 years, 365 (88.8%) were married, 223 (54.3%) had secondary education, 185 (45.3%) were retired, and 124 (34.3%) had a monthly income 2001–4000 RMB (Chinese Renminbi about US$140). There was no significant difference in demographic information between those who had quit smoking and those who did not (p>0.05). The demographic information is shown in Table 1.

**Table 1:** Demographic characteristics of a community smoking cessation intervention project in Beijing, 2018–2019 (N=412)

*Characteristics*	*Quitters n (%)*	*Non-quitters n (%)*	*p*
**Gender**			
Male	143 (38.2)	231 (61.8)	0.641
Female	16 (42.1)	22 (57.9)	
**Age** (years)			
20–29	3 (25.0)	9 (75.0)	0.164^a^
30–39	16 (30.8)	36 (69.2)	
40–49	23 (45.1)	28 (54.9)	
50–59	41 (32.8)	84 (67.2)	
60–69	60 (43.2)	79 (56.8)	
≥70	16 (50.0)	16 (50.0)	
**Marital status**			
Unmarried	3 (13.6)	19 (86.4)	0.060^a^
Married	148 (40.5)	217 (59.5)	
Divorced	4 (28.6)	10 (71.4)	
Widowed	4 (40.0)	6 (60.0)	
**Education level**			
Primary or lower	8 (34.8)	15 (65.2)	0.861
Secondary	85 (38.1)	138 (61.9)	
Tertiary	66 (40.0)	99 (60.0)	
**Employment status**			
Unemployed	3 (33.3)	6 (66.7)	0.101^a^
Employed	73 (34.1)	141 (65.9)	
Retired	82 (44.3)	103 (55.7)	
**Monthly income** (RMB)			
≤2000	19 (46.3)	22 (53.7)	0.492
2001–4000	45 (36.3)	79 (63.7)	
4001–6000	42 (45.7)	50 (54.3)	
6001–8000	13 (30.2)	30 (69.8)	
8001–10000	10 (37.0)	17 (63.0)	
>10000	14 (40.0)	21 (60.0)	

a Fisher exact probability test.

Some sociodemographic information was missing: age (n=1), marital status (n=1), education level (n=1), work status (n=4), and monthly income (n=50). RMB: Chinese Renminbi about US$140.

### Community smoking cessation intervention

To ensure comparability of data on the environment and social support for tobacco control between quitters and non-quitters, we excluded the effect of previous study interventions. In a previous study^18^, there were 159 (38.6%) participants in the control group and 253 (61.4%) in the intervention group (the intervention follow-up cohort). There was no significant difference in the effect of tobacco control intervention between those who had quit and those who did not quit (p>0.05). The specific intervention plan is described elsewhere^15,18^. The community smoking cessation intervention group comparisons are shown in Tables 2 and 3.

**Table 2:** Smoking cessation outcome in different groups of a community smoking cessation intervention in Beijing, 2018–2019 (N=412)

*Groups*	*Quitters n (%)*	*Non-quitters n (%)*	*χ^2^*	*p*
Control	52 (32.7)	107 (67.3)	3.788	0.052
Intervention	107 (42.3)	146 (57.7)		

**Table 3:** The 6-month smoking reduction in different groups of a community smoking cessation intervention in Beijing, 2018–2019 (N=412)

*Groups*	*n*	*%*	*Smoking reduction Median (IQR)*	*U*	*p*
Control	159	38.6	9 (9–10)	18098.5	0.074
Intervention	253	61.4	9 (5–10)		

IQR: interquartile range.

### Environment and social support of tobacco control

We conducted a chi-squared test and Fisher’s exact test to determine the correlation between the variables and smoking cessation. Overall, 375 (91.0%) of the participants were daily smokers, and 334 participants (81.1%) reported that tobacco regulations existed in their community, home, and workplace; 220 participants (57.4%) had seen smoking in indoor places in their community; 320 participants (84.4%) had seen smoking in outdoor places in their community; and 304 participants (73.8%) were able to learn about the hazards of tobacco through community publicity, with community bulletin boards, billboards, and printed products being the main means of access; 119 participants (29.8%) reported that smoking did not occur in their home every month or never occurred; and 211 participants (52.5%) had strong family support for smoking cessation. On average, there were 1.37 ± 0.63 smokers in the family, 159 participants (38.6%) had quit smoking, and the average 6-month reduction in smoking for all 412 participants was 8.96 ± 8.78 cigarettes.

The results were significantly different between home tobacco regulations, workplace tobacco regulations, and the number of smokers in the family (p<0.05). With the clarification and strictness of home tobacco regulations, the rate of smoking cessation will increase; in the workplace, a gradual increase in the strictness of tobacco regulations will also have a positive effect on rate of smoking cessation among smokers. In addition, the rate decreases with the increase in the number of smokers in the family, the smokers in the family being critical to the rate of smoking cessation.

Results of the univariate analysis are shown in Table 4. The results of the non-parametric test (Kruskal-Wallis test) show that 6-month smoking reduction is significantly associated with anti-tobacco messaging and the frequency of smoking events at home (p<0.05). The results of the non-parametric test for 6-month smoking reduction are shown in Table 5.

**Table 4:** Differences in environment and social support variables for tobacco control on smoking cessation outcome (N=412)

*Variables*	*Quitters n (%)*	*Non-quitters n (%)*	*χ^2^*	*p*
**Home tobacco regulations**				
Not allowed anywhere in the home	44 (50.0)	44 (50.0)	8.206	0.017
Not allowed in some places or sometimes	45 (40.9)	65 (59.1)		
No regulations	70 (32.7)	144 (67.3)		
**Workplace tobacco regulations**				
Not allowed at all	55 (46.6)	63 (53.4)	11.189	0.011
Allowed in smoking areas	35 (37.2)	59 (62.8)		
No regulations	19 (24.7)	61 (76.3)		
Not hired or not known	50 (41.7)	70 (58.3)		
**Number of smokers in the family**				
1	126 (42.9)	168 (57.1)	9.351	0.016^a^
2	24 (25.5)	70 (74.5)		
3	8 (40.0)	12 (60.0)		
≥4	1 (25.0)	3 (75.0)		

a Fisher’s exact probability test.

**Table 5:** Differences in environment and social support variables for tobacco control on 6-month smoking reduction (N=412)

*Variables*	*n*	*%*	*Smoking reduction Median (IQR)*	*H_c_ value^a^*	*p*
**Exposure to anti-tobacco messaging** (number of ways)					
0	108	26.2	9 (6.25–9.75)	16.328	0.006
1	98	23.8	9 (9–10.75)		
2	102	24.8	9 (4.75–9)		
3	52	12.6	9 (9–10)		
4	21	5.1	9 (9–10)		
5	31	7.5	9 (9–12)		
**Frequency of smoking events at home**					
Daily	239	58.0	9 (9–10)	11.737	0.039
Weekly	25	6.1	9 (0–9)		
Monthly	17	4.1	9 (0.5–9.5)		
Don’t know/don’t remember	31	7.5	9 (7–9)		
Not every month	88	21.4	9 (7.25–9.75)		
Never	12	2.9	9 (0–9)		

a Kruskal-Wallis test. IQR: interquartile range.

Binary logistic regression analysis was performed to clarify the relationship between variables and smoking cessation. The results show that the factors affecting smoking cessation outcomes included home tobacco regulations, workplace tobacco regulations, and the frequency of smoking events at home (p<0.05). A higher level of stricter home tobacco regulations increased the likelihood of smokers smoking cessation success by 1.302 times; and a higher level of stricter workplace tobacco regulations increased the likelihood of smokers smoking cessation success by 1.273 times. In terms of the frequency of smoking events at home, the lower the frequency, the more likely the smoker had smoking cessation success by 1.145 times. This shows that the strictness of tobacco regulations in the smoker’s living environment and the frequency of smoking behavior in the home has a significant effect on smoking cessation outcome and positively influences the rate of smoking cessation. The binary logistic regression analysis of smoking cessation outcome is shown in Figure 1.

### Structural equation modeling

A structural equation model was used to verify and supplement the binary logistic regression analysis. The set of variables and structural equation model were constructed based on the survey data, correlation coefficient matrix, and model fitting information. Results of the fitness test of the SEM of smoking cessation outcomes, show the SEM has a good fit (Supplementary file Table S2).

Environment support was explained by home tobacco regulations (β=0.49; 95% CI: 0.25–0.73, p<0.001), workplace tobacco regulations (β=0.48; 95% CI: 0.25–0.70, p<0.001) and anti-tobacco messaging (β=0.29; 95% CI: 0.11–0.47, p=0.001). Social support was explained by the frequency of smoking events at home (β=0.70; 95% CI: 0.36–1.04, p<0.001), and number of smokers in the family (β= -0.55; 95% CI: -0.84 – -0.27, p<0.001). Smoking cessation was explained by smoking cessation outcome (β=0.93; 95% CI: 0.30–1.55, p=0.004), 6-month smoking reduction (β=0.26; 95% CI: 0.07– 0.45, p=0.008), environment support (β=0.39; 95% CI: 0.05–0.73, p=0.026) and social support (β=0.37; 95% CI: 0.05–0.68, p=0.022).

**Figure 2:**
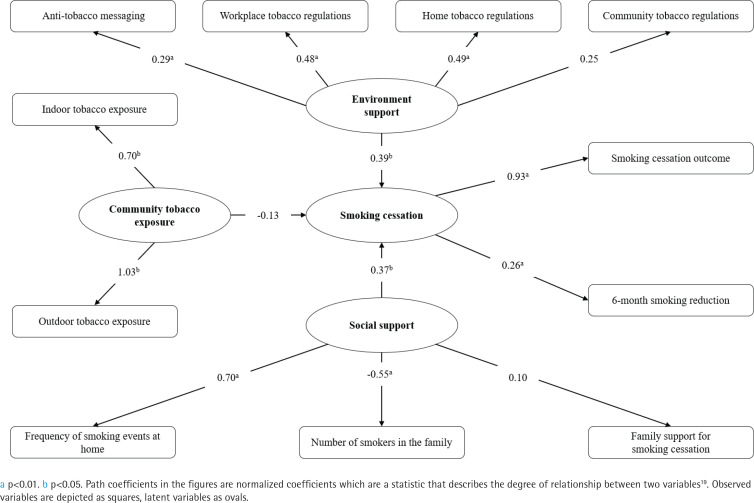
Results of structural equation modeling for smoking cessation, 2019 (N=375)

The stricter the regulations on tobacco at home and in the workplace, and the more ways for anti-tobacco messaging, the higher the rate of smoking cessation. The lower the frequency of smoking at home and the fewer the smokers in the family, the higher the rate of smoking cessation. The results of the structural equation model are shown in Figure 2.

## DISCUSSION

Tobacco dependence is recognized as a chronic disease by the World Health Organization (WHO), but perseverance to quit smoking without outside intervention has only about a 3% chance of success. Only pharmacotherapy combined with behavioral interventions, psychotherapy, environmental and social support can free most smokers from tobacco dependence^20-22^. Similarly, we found that environmental support and social support are associated with smoking cessation. Attention should be paid to the smoking regulations at home and workplace, anti-tobacco messaging, and social support in the family.

### Environment support

The results of binary logistic regression analysis and the SEM in this study suggest that the stringency of tobacco regulations in the smokers’ living environment and the publicity of anti-tobacco messaging are positively associated with rate of smoking cessation. The setting of tobacco regulations in smokers’ living places has a positive effect on smoking cessation attempts and smoking cessation outcomes, and strict tobacco regulations can restrain smokers’ smoking behavior in different places; at the same time, tobacco regulations can guide smoking cessation attempts and enhance the cessation outcome. Zhang et al.^23^ showed that smoking cessation attempt rate with tobacco regulations at home was 26.0%, compared to 12.6% without relevant regulations at home; those with strict tobacco regulations at home were 2.395 times more likely to try to quit smoking than those who were allowed to smoke at home; those with strict tobacco regulations in their workplace were 1.657 times more likely to try to quit smoking than those without regulations. Soule et al.^24^ showed that tobacco regulations can have some effects on smoking cessation, and tobacco regulations may increase smoking cessation attempts. Therefore, the community should strengthen the dissemination of family tobacco control regulations, promote smoke-free living, and ultimately improve smoking cessation outcomes.

### Exposure to anti-tobacco messaging

The results show that there are differences in the effectiveness of smoking cessation among people who receive anti-tobacco messaging through different ways. Most smokers obtained anti-tobacco messaging by means of community bulletin boards and billboards. This result is consistent with the findings of Zhao^25^ that smokers can obtain anti-tobacco messaging through different ways. Just as Lu et al.^26^ showed that 45.1% had seen tobacco control messages in newspapers/magazines in the past 30 days, 89.9% of smokers had seen smoking cessation warnings on cigarette packages, but only 20.0% of smokers would consider quitting smoking as a result. Meanwhile, Qian et al.^27^ showed that online smoking cessation community can bring quitters together. Users provide each other with smoking cessation support and improve smokers’ compliance to quit. Thus, smokers have limited access to anti-tobacco messaging, most of which is focused on a single pathway, and it is ineffective in promoting smoking cessation. We should thus broaden the ways of tobacco harm publicity and improve the publicity effect, strengthen the combination of anti-tobacco messaging and network information platforms to publicize anti-tobacco messaging. It is important that smokers obtain anti-tobacco messaging and smoking cessation experience through various ways.

### Social support

The results showed that the number of smokers in the family and the frequency of smoking events at home are key factors in enhancing smoking cessation, and the results of the SEM were consistent with these results. The higher the frequency of smoking events at home or large number of smokers in the family both lead to frequent contact with smoking behaviors and smoking-related people for those who are trying to quit, creating visual triggers for craving of cigarettes^28^. Okechukw et al.^29^ stated that partner smoking in the family is a significant predictor of smoking behavior among smokers. Pereira et al.^30^ reported that 53.2% of smokers had other members in their family who smoked, significantly higher than those who had quit smoking and those who never smoked. In the study of Xian and Wang^21^, contact with people or places associated with past smoking was suggested as a trigger for relapse in quitters. Therefore, it is suggested that we should focus on the establishment of home tobacco regulations to inform visitors that smoking is prohibited in the home, to reduce exposure to smoking behaviors for those who are in the process of smoking cessation.

### Strength and limitations

This study ascertained the factors of the environment and social support variables for tobacco control and analyzed the mechanisms and extent of influence factors on the smoking cessation outcome. Most previous studies regarded environment or social support as a theme in the community, focusing on the distribution of multiple themes, while our study focused on environment and social support separately. However, the study has limitations. Firstly, this study used only two cross-sectional data from the baseline survey and the sixth month survey, and did not use continuous, long-term follow-up data. Secondly, the study site was Beijing, and cities without tobacco control legislation were not selected as a control group. Thirdly, the survey of the number of smokers in the family did not take into account whether the subjects themselves were excluded, which may have led to bias in the number of smokers in the family. Fourthly, there is recall bias, smokers who had quit smoking may be more aware of anti-tobacco influences in their environment simply because they have recently been focused on quitting.

## CONCLUSIONS

In this study, the environment and social support variables for tobacco control were divided into three aspects: environment support, community tobacco exposure, and social support. Results showed that home tobacco regulations, workplace tobacco regulations, anti-tobacco messaging, and the frequency of smoking events at home positively influenced smoking cessation outcomes, while the number of smokers in the family negatively influenced smoking cessation. There is a need for establishing tobacco regulations in the community, home, workplace and other living places, increasing anti-tobacco messaging and building social support to promote great concern in society, and interventions concerning those factors should be undertaken.

## Data Availability

The data supporting this research are available from the authors on reasonable request.
